# Self-Healing, Robust, Liquid-Repellent Coatings Exploiting the Donor–Acceptor Self-Assembly

**DOI:** 10.1021/acsami.2c20636

**Published:** 2023-02-03

**Authors:** Jianhui Zhang, Vikramjeet Singh, Wei Huang, Priya Mandal, Manish K. Tiwari

**Affiliations:** † Nanoengineered Systems Laboratory, UCL Mechanical Engineering, University College London, London WC1E 7JE, U.K.; ‡ Wellcome/EPSRC Centre for Interventional and Surgical Sciences, University College London, London W1W 7TS, U.K.

**Keywords:** liquid-repellent coating, donor−acceptor self-assembly, metal−organic frameworks, rapid self-healing, substrate adhesion, anti-icing

## Abstract

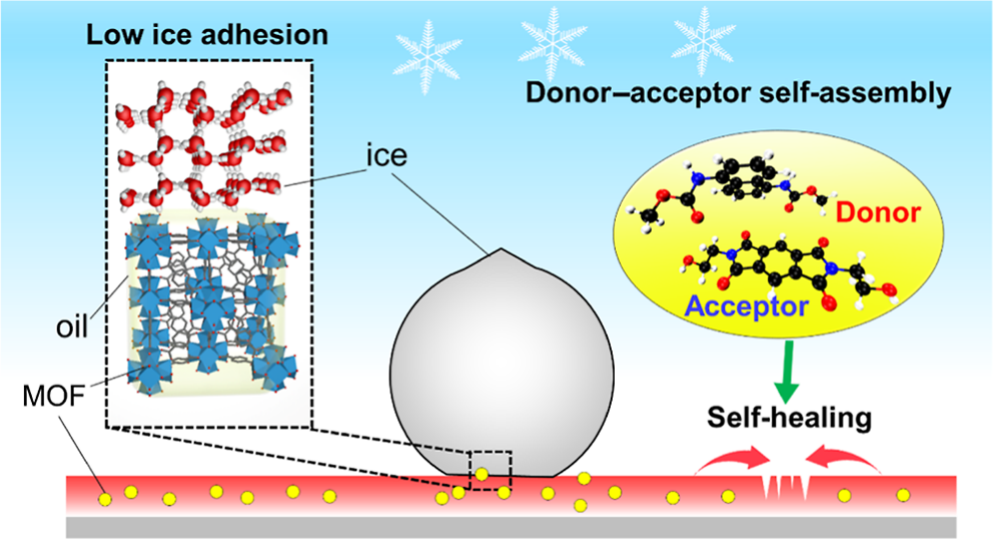

Liquid-repellent coatings with rapid self-healing and strong substrate adhesion have tremendous potential for industrial applications, but their formulation is challenging. We exploit synergistic chemistry between donor–acceptor self-assembly units of polyurethane and hydrophobic metal–organic framework (MOF) nanoparticles to overcome this challenge. The nanocomposite features a nanohierarchical morphology with excellent liquid repellence. Using polyurethane as a base polymer, the incorporated donor–acceptor self-assembly enables high strength, excellent self-healing property, and strong adhesion strength on multiple substrates. The interaction mechanism of donor–acceptor self-assembly was revealed via density functional theory and infrared spectroscopy. The superhydrophobicity of polyurethane was achieved by introducing alkyl-functionalized MOF nanoparticles and post-application silanization. The combination of the self-healing polymer and nanohierarchical MOF nanoparticles results in self-cleaning capability, resistance to tape peel and high-speed liquid jet impacts, recoverable liquid repellence over a self-healed notch, and low ice adhesion up to 50 icing/deicing cycles. By exploiting the porosity of MOF nanoparticles in our nanocomposites, fluorine-free, slippery liquid-infused porous surfaces with stable, low ice adhesion strengths were also achieved by infusing silicone oil into the coatings.

## 1 Introduction

Liquid-repellent surfaces, including superhydrophobic surfaces (SHS) and slippery liquid-infused porous surfaces (SLIPS), have the potential to improve anti-icing, water collection, and antifouling properties remarkably for industrial applications.^1−4^ Although there are several fabrication methods and materials, their robustness and durability^5−9^ leave a huge scope for improvement. To develop robust liquid-repellent surfaces, the combination of abrasion-resistant materials and elastic supporting polymers is commonly applied to reduce the damage to the surface passively.^7,10^ Generally, coatings with low surface energy adhere poorly to most substrates, with a few specific exceptions.^11,12^ Even with high substrate adhesion, surfaces are often exposed to high-speed liquid impact, mechanical abrasion, chemical degradation, and so forth.^5,7^ Such challenging conditions can easily exceed the inherent strength and toughness of most materials. Hence, it is desirable to incorporate self-healing properties to help the surfaces recover from mechanical damage and thus prolong their durability.^13−17^ A major hurdle to self-healing ability in most materials is the requirement of external stimuli with high temperatures (even > 100 °C) and/or long recovery times (∼10^2^ to 10^3^ min), thus limiting their practical applications.^14,18^ For example, a recently developed polydimethylsiloxane (PDMS)-based SHS, crosslinked by dynamic hydrogen bonds and coordination bonds, required high-temperature heating (120 °C) for healing within minutes from physical damages.^19^ The combination of rapid self-healing (<10 min) SHS with high substrate adhesion remains a challenge. This requires at least two sets of advances: the first around the self-healing polymer matrix and the second around an appropriate choice of filler nanoparticles, which can enable robust self-healing while enhancing the liquid repellence. Here, we introduce donor–acceptor (D–A) self-assembly into a standard polyurethane matrix and metal–organic framework (MOF) nanoparticles to address these key requirements.

The reversible and repeatable self-healing capability of polymer materials is usually achieved by introducing repairable chemical bonds or noncovalent interactions.^20−22^ Over the past decade, self-assembled supramolecular polymers^23^ have exploited noncovalent interaction, such as aromatic π–π stacking,^24^ metal–ligand interactions,^25^ hydrophobic effects,^26^ and so forth, to achieve rapid self-healing in response to stimuli and adjustability of tensile strength. Due to the low surface energy of organosilicon molecular chains, PDMS-based supramolecular polymers have been developed for self-healing SHS through mechanisms, including N-coordinated boroxines,^27^ dynamic hydrogen bonds, and metal–ligand coordination bonds.^19^ However, due to the limited migration and mobility of polymer chains, high-temperature stimuli are still required to heal polymer from damages.^19^ Recently, a polyurethane (PU) based on donor–acceptor self-assembly (DA-PU) with remarkable thermal reparability and self-healing capabilities was reported.^28^ In comparison to other noncovalent interactions, the interaction between the naphthalene ring (as donor) and the imide group (as acceptor) in DA-PU enables greater toughness and faster thermally activated healing properties. Self-assembly of D–A inter- and intrachain may help improve the microstructure of polymeric materials, essentially following a mechanism that mimics the reversible breakage and refolding of secondary interactions within the skeletal muscle proteins which induce recovery after damage.^29^ Through interchain self-assembly, polymers with electron donors or acceptors units at the chain ends have also been reported to achieve markedly improved mechanical characteristics and self-healing capability.^24^ However, the enhancement of PU adhesion strength, which is due to a known feature of the aromatic group stacking during D–A self-assembly,^30,31^ has not been investigated. Furthermore, despite its attractive properties, nonwetting surfaces based on this PU have also not been investigated.

In the current work, we crucially introduce a number of notable advances by incorporating MOF nanoparticles in this polymer. First, the mechanism of D–A self-assembly featuring a hierarchy of multiple hydrogen bonds, and the overlap of the associated electrostatic potential (ESP) surfaces was investigated by density function theory (DFT) and confirmed by Fourier transform infrared spectroscopy (FTIR). Ultrafast self-healing (<1 min) of a notched DA-PU film was achieved by modest heating at 80 °C. In addition to the excellent mechanical properties with elongation at break of 818% and toughness of 132.8 MJ/m^3^, DA-PU also showed remarkable universal adhesive properties on different substrates, such as glass, metals (e.g., copper and aluminium), and even polytetrafluoroethylene (PTFE). These excellent mechanical, interfacial adhesion, and self-healing properties emerge from the stacking molecular conformation of the D–A complex, which has a low energy barrier (−4.52 kcal/mol) for the self-assembly. Second, fluorine-free hydrophobic nanohierarchical MOF nanoparticles with remarkable thermochemical stability and scratch resistance were incorporated into DA-PU to introduce a robust nanohierarchical texture^32^ for SHS. A hydrophobic alkyl-functionalized zirconium-based MOF (alkyl-UiO-66) was synthesized using known techniques,^33^ and the superhydrophobic coatings were fabricated by spraying a solution of DA-PU and MOF nanoparticles, followed by silanization. Only this zirconium-based MOF was used in this work due to its hydrolytic, thermochemical, and mechanical stability.^33^ A systematic consideration of different MOF particles is beyond the scope of the current work. The fine surface asperities of MOF nanoparticles and the flexibility of the polymer hosted in the resulting nanocomposite coating are expected to lend robustness and impalement resistance to the SHS.^7,32,34^ Resistance to high-speed liquid impact (up to ∼35 m/s, Weber number ∼ 42,500) and low ice adhesion were confirmed for the as-prepared coatings. Lastly, by exploiting the high porosity of MOF in the coatings, SLIPS could be prepared simply by infusing silicone oil into the SHS.^35^ Benefiting from the nanohierarchical texture and/or the enhanced interaction between functionalized MOF nanoparticles and the lubricant, both SHS and SLIPS show low ice adhesion over 50 icing/deicing cycles, which should have widespread uses in low-temperature applications.^36^

## 2 Material and Methods

### 2.1 Materials

Hexamethylene diisocyanate (HDI), polycaprolactone diol (PCL-OH, Mn ∼ 2000 g mol^–1^), pyromellitic dianhydride (PMD), dibutyltin dilaurate (DBTDL), ethanolamine, zirconium oxychloride octahydrate (ZrCl_2_·8H_2_O), 2-aminoterepthalic acid (ATPA), octanoic acid, sodium hydroxide, acetic acid, trichloro(octadecyl)silane (OTS), dimethyl formamide (DMF), chloroform, acetone, toluene, isopropanol, hexane, and silicone oil (20, 100, 500, and 1000 cSt) were purchased from Sigma-Aldrich. 1,5-Diisocyanatonaphthalene (NDI) was purchased from Tokyo Chemical Industry UK Ltd. All the chemicals were used without further purification. Commercial NeverWet coating components, used as a control for comparison, were obtained from the Rust-Oleum.

### 2.2 Instrumentations and Characterization

Surface morphologies were imaged using a scanning electron microscopy (SEM) (EVO25, Carl Zeiss, Germany), following a thin gold film sputtering, at 5 kV voltage and 5 pA current. FTIR spectra was recorded with a spectrophotometer (Spectrum Two, PerkinElmer, ATR mode). A digital microscope (Keyence, VHX-7000) and a GXML3200B compound microscope were used to observe the self-healing process and examine the damage to coatings following liquid impact tests. The powder X-ray diffraction (PXRD) of the MOF powder was recorded on a Stoe STADI-P spectrometer with tube voltage of 40 kV, tube current of 40 mA in a stepwise scan mode (5° min^–1^). The transparency was assessed using an Orion AquaMate UV–vis spectrophotometer. The contact angles and the contact angle hysteresis were measured on a custom designed goniometer setup with at least three samples tested for statistics.^32^

### 2.3 Synthesis of DA-PU

A method reported by Ying et al.^28^ was adopted to synthesize DA-PU. PMD (0.104 g) and ethanolamine (0.058 g), dissolved in 10 mL DMF, were mixed and heated at 150 °C for 1 h to synthesize the acceptor unit of DA-PU (PMDA-OH). The prepolymerization reaction of PU with the donor unit was performed in a N_2_ glove-box. PCL-OH (0.952 g), NDI (0.100 g), HDI (0.080 g), and DBTDL (0.001 g) were added into glass bottle containing a magnetic stirrer, followed by the addition of 4.526 g DMF and heating in a 60 °C oil bath for 1 h. After prepolymerization, the acceptor unit PMDA-OH was added in for chain extension, and the mixture was heated at 80 °C (in oil bath) for 5 h to complete the polymerization reaction of DA-PU. The polymer was then precipitated using excess deionized water and washed five times to remove the impurities and organic solvents. After that, the raw DA-PU was dried overnight in vacuum oven at 80 °C. The synthesis method of PU was similar to the prepolymerization reaction of DA-PU, except that no donor (NDI) and acceptor unit (PMDA-OH) were added, and the stoichiometric ratio of [NCO]/[OH] was used. The PU polymerization was performed by heating at 80 °C for 6 h, followed by overnight drying in vacuum oven at 80 °C to obtain the polymer in the form of a sedimented film.

### 2.4 Synthesis of Hydrophobic MOF

Hydrophobic MOF (alkyl-UiO-66) was synthesized through the typical condensation reaction between amino functional groups of NH_2_-UiO-66 and carboxylic acid functional groups of octanoic acid. An ambient-temperature aqueous synthesis was adopted for NH_2_-UiO-66.^37^ ATPA (0.181 g) and sodium hydroxide (0.08 g) were dissolved in 5 mL of deionized water at 60 °C for 10 min and then cooled to room temperature to obtain the sodium salt of the organic linker. Next, ZrOCl_2_·8H_2_O (0.322 g) and acetic acid (100 mM) were solubilized in 5 mL of deionized water. A yellow precipitate was obtained by slowly adding one of above-prepared solutions into another dropwise. The mixture was stirred for 12 h at room temperature to complete the MOF synthesis reaction. The obtained crystals were centrifuged and washed 3 times with deionized water. Lastly, the trapped water in pores was removed with the Soxhlet method in ethanol for 16 h at 120 °C, and the resulting crystalline powder was dried overnight at 150 °C under vacuum. The pores of NH_2_-UiO-66 were modified through straightforward postsynthesis functionalization to obtain hydrophobic alkyl-UiO-66. To do this, the dried NH_2_-UiO-66 powders were dispersed in DMF, and 1 equivalent of octanoic acid to organic linker (APTA) was added into the solution and stirred at 80 °C for 12 h. After that, the crystals were centrifuged and washed with DMF and acetone twice and then in chloroform thrice, followed by drying overnight under vacuum at 120 °C. The successful synthesis of alkyl-UiO-66 was confirmed by FTIR spectroscopy (Figure S6a) and PXRD (Figure S7 in Supporting Information).

### 2.5 Fabrication of SHS and SLIPS

Glass slides of 2.5 cm × 7.5 cm were used as substrates. The slides were cleaned with acetone and isopropanol, followed by nitrogen purging. Hydrophobic MOF nanoparticles were added to acetone and sonicated for 30 min to get a well-dispersed suspension. DA-PU (0.1 g) and PU (0.1 g) as controls were dissolved in MOF suspension, respectively. The weight ratio between MOF nanoparticles and DA-PU or PU was kept at 1:1, which was determined by preparing coatings with different MOF nanoparticle loadings and testing hydrophobicity (Figure S9). The mixture solution was sprayed with a spray gun (Iwata Eclipse, ECL2000) at 2.5 bar pressure. The weight of coating on each glass slide was kept ∼55 mg. After spraying, the samples were dried overnight in a vacuum at 60 °C. Lastly, the samples were placed into a Petri dish with 20 mL hexane and 50 μL OTS for 2 h, followed by heating the samples at 100 °C for 30 min to achieve superhydrophobicity.

SLIPS were fabricated by infusing silicone oil into the DA-PU/MOF SHS. The oil infusion was achieved using thermal diffusion at 80 °C for 12 h followed by cooling to room temperature. After infusion, the samples were spun at 2000 rpm to get rid of excessive lubricant. All surfaces (2.5 cm × 7.5 cm) were then subjected to contact angle, tape peel, and self-healing tests (see Supporting Information) and ice adhesion measurement, as explained below.

### 2.6 Ice Adhesion Measurement

Ice adhesion measurements were performed by placing the samples in a custom designed bench-top icing chamber, as reported previously.^32^ A schematic of the ice adhesion measurement is shown in Figure 6e. Bare glass with an area of 2.5 cm × 7.5 cm and commercial superhydrophobic coatings (NeverWet) on glass were used as control samples. After the temperature inside the chamber reached −20 °C, water was poured into cuvettes with a base area of 1 cm × 1 cm and frozen for 1 h. A rod connected to a force gauge was pushed laterally against the cuvettes at a low speed. The peak force required to remove the ice was noted. Ice adhesion strength was defined as the maximum force required to remove ice per unit area. The average ice adhesion strength was obtained from at least three independent measurements on different samples.

## 3 Results and Discussion

### 3.1 Polyurethane with Donor–Acceptor Self-Assembly

**Figure 1:**
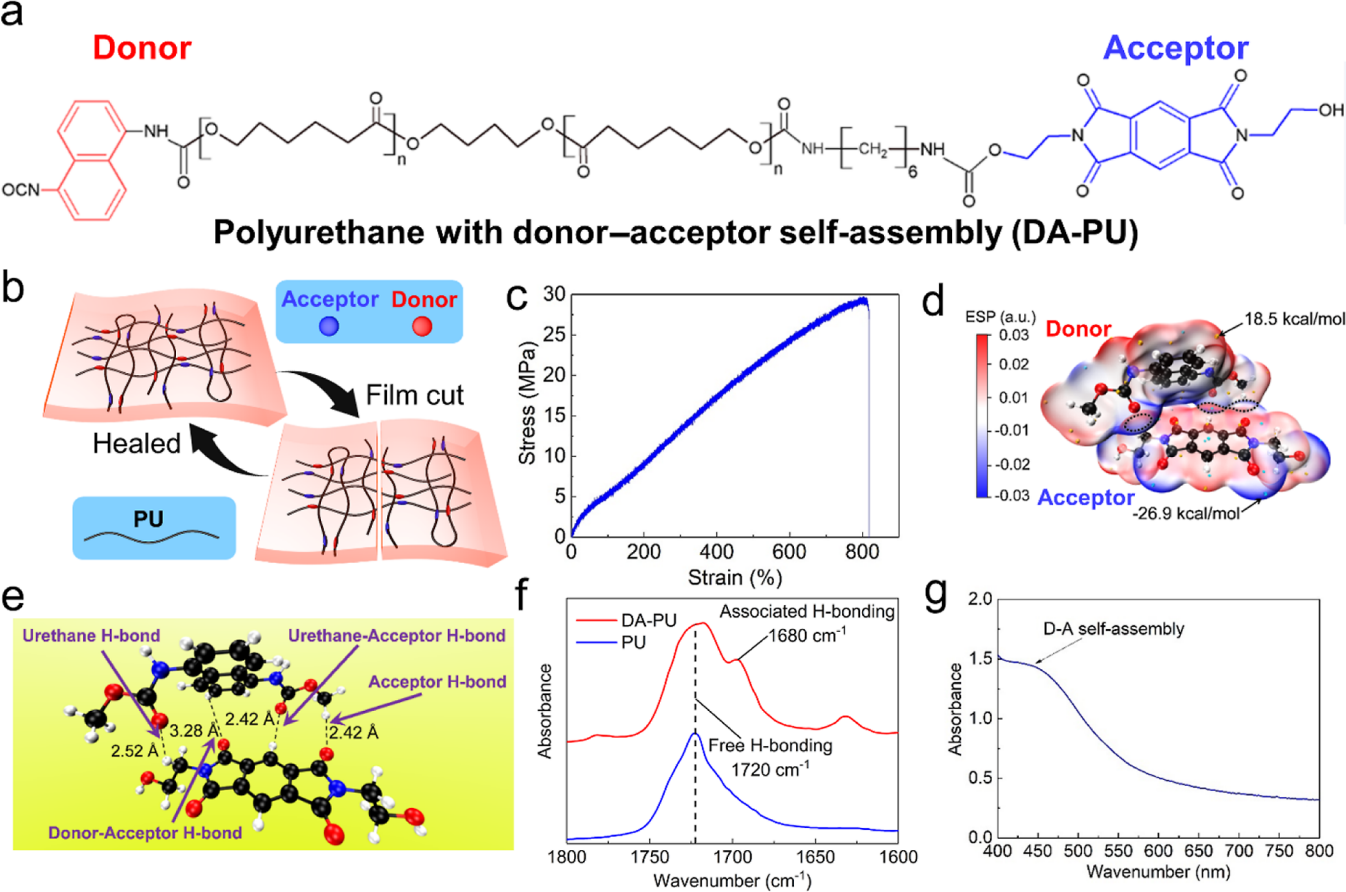
Molecular structure, DFT calculation, and infrared spectroscopy of DA-PU. (a) Chemical structure of DA-PU. (b) The schematic illustration of the self-healing process with inter- and intra-chain interactions. (c) Stress–strain curve of DA-PU (standard dumbbell shape specimen, ASTM D412, measured in Instron, model 5969 at a constant speed of 1 mm/min). (d) ESP of the D–A complex. The negative surface potential is indicated in blue, while the positive surface potential is indicated in red. The dotted lines mark the overlapping parts of the two van der Waals surfaces, which are colored by ESP. Gold and cyan dots correspond to surface maxima and minima, respectively. (e) Molecular structure of the D–A complex after optimization and its multiple hydrogen bonds. (f) FTIR spectra of DA-PU and PU, showing the peak at 1680 and 1720 cm^–1^ corresponding to the associated and free hydrogen bonds, respectively. (g) UV–vis spectra of DA-PU dissolved in DMF at the concentration of 30 g/L. The characteristic peak, at 450 nm, proves the existence of interchain D–A self-assembly.^28^

Figure 1a shows the molecular structure of DA-PU, with donor (D) and acceptor (A) units distributed along the polyurethane (PU) chain. The conceived synthesis route for DA-PU is provided in Figure S5. The noncovalent interaction with both intra- and interchain self-assembly (as shown in Figure 1b) enable the PU extraordinary performance, including enhancement of adhesive, self-healing, and excellent mechanical properties (e.g., elongation at break: 818% and toughness: 132.8 MJ/m^3^, see Figure 1c). As opposed to the ref (28), we replaced polytetramethylene ether glycol (PTMEG) in the DA-PU soft segment with crystallizable polycaprolactone diol (PCL).^38^ This result in an increased tensile strength of DA-PU from reported 25^28^ to 30 MPa (Figure 1c). DFT and ESP analysis was employed to study the interaction between D and A units (see in the Supporting Information). The simulation results indicate that the surface of naphthalene from donor is positively charged, while the dianhydride group is the negatively charged (Figure 1d; Figure S1, Supporting Information). The three overlapping zones in the van der Waals surfaces for D and A unit (demarcated by dotted lines) attest to strong electrostatic attraction and charge-transfer complex formation^39^ between them. In Figure 1d, the surfaces are plotted using an ESP-based color map. The interaction energy between D and A units was calculated as −4.52 kcal/mol by the counterpoise correction method.^40^ A hierarchy of four different types of hydrogen bonds in D–A complex should increase the strength of the hard phase from polyurethane microphase structure (Figure 1e), and the existence of these hydrogen bonding associations was proved by FTIR (Figures 1f and S6b). The existence of interchain D–A self-assembly was also manifested in the UV–vis spectra, featuring a characteristic peak around 450 nm (Figure 1g).^28^

### 3.2 Rapid Thermal Self-Healing

**Figure 2:**
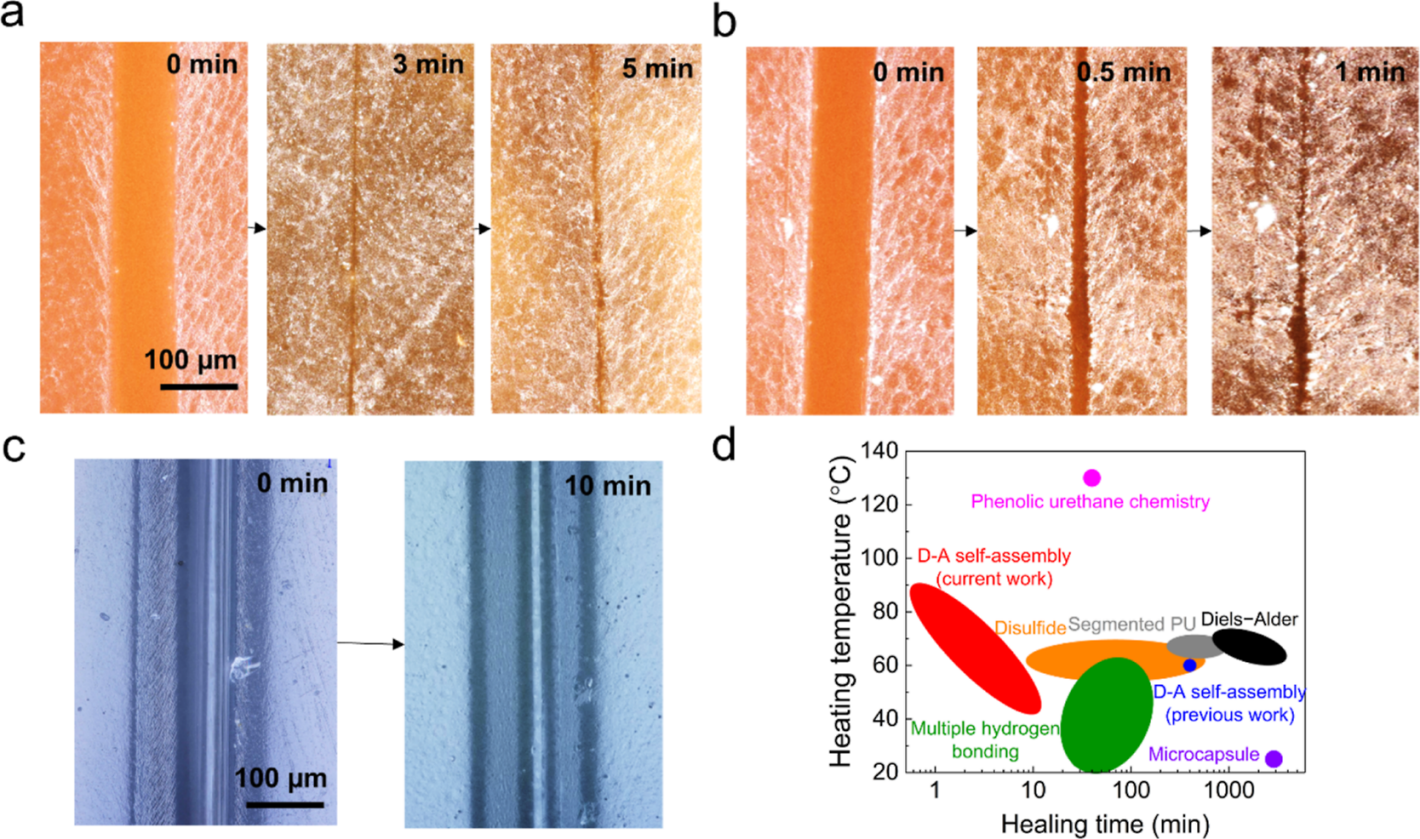
Self-healing properties of the notched DA-PU and PU films. (a) Optical microscope images of the notched DA-PU (left, 0 min) and healed films after 3 and 5 min (right) at 60 °C. (b) Optical microscope images of the notched DA-PU film showing fast disappearance of the scar during self-healing at 80 °C for 1 min. (c) Optical microscope images of the notched PU film showing slowly healing of the scar at 80 °C for 10 min. Scale bar: 100 μm. (d) Comparison of the self-healing properties of DA-PU with different repair mechanisms. (Previous work on D–A self-assembly,^28^ Diels–Alder chemistry,^14,44^ disulphide linkage,^45−47^ phenolic urethane chemistry,^18^ microcapsule,^48^ segmented PU,^49^ and multiple hydrogen bonding.^50−53^ Details are presented in Table S1 in the Supporting Information).

Figure 2a presents the self-healing process of DA-PU at 60 °C (see Methods in the Supporting Information). At first, a notch about 100 μm wide and 400 μm depth divided the sample into left and right parts. The notch disappeared almost completely with a much narrower trace left after 3 min, and no discernible changes were observed after 5 min. When the temperature was increased to 80 °C, DA-PU healed quickly within 1 min (in Figure 2b). As a comparison, the notch of common PU in Figure 2c showed little change in 10 min at 80 °C, indicating that the hydrogen bonding associations during D–A self-assembly had a stronger effect on self-healing than just hydrogen bonds in urethane links of PU. The in situ self-healing dynamic process was also recorded with a Peltier plate under the sample (see Movie S1). A Peltier plate (heating from bottom) induced a bottom-to-top self-healing process due to inevitable thermal gradient, which was different from the uniform healing (marked by cut interfaces approaching each other) under uniform temperature field of oven heating shown in Figure 2a,b. DA-PU could not heal at room temperature; however, efficient self-healing (<15 min) could be achieved with a small thermal stimulus (40–60 °C) (Figure S2 in the Supporting Information). Furthermore, we compared the self-healing properties of our DA-PU with the other reported functionalized PU in Figure 2d and Table S1. The previously reported DA-PU was an elastomer containing PTMEG, while others were mainly used as functional coating. Compared with PTMEG-based DA-PU, the soft segment with higher molecular weight, PCL-OH, applied in this work could increase the glass transition temperature and molecular chain mobility of DA-PU during heating, leading to the better self-healing performance.^41^ Among those reported PU, our DA-PU shows remarkable self-healing properties with shortest healing time.

The DFT was used to decipher the atomic scale underpinnings of the D–A self-assembly and its role in self-healing. In the literature, broadly, either disulfide or hydrogen bonding are exploited to introduce self-healing characteristics in a polymer matrix.^42^ Due to the high energy barrier (57–91 kcal/mol) of thermodynamic exchange reactions in disulfide linkage^43^ and microphase separation induced by hydrogen bond dissociation, both generally require high temperatures in the range of 60–110 °C. A much weaker interaction energy was calculated between the donor and acceptor (−4.52 kcal/mol) using DFT simulation, which explains the excellent self-healing at relatively lower temperature that we observed. Overall, D–A interaction forms special hierarchal hydrogen bonds induced by strong electrostatic attraction and lamellar molecules stacking, resulting in rapid self-healing and high surface adhesion, as shown in next section.

### 3.3 Adhesive Properties

**Figure 3:**
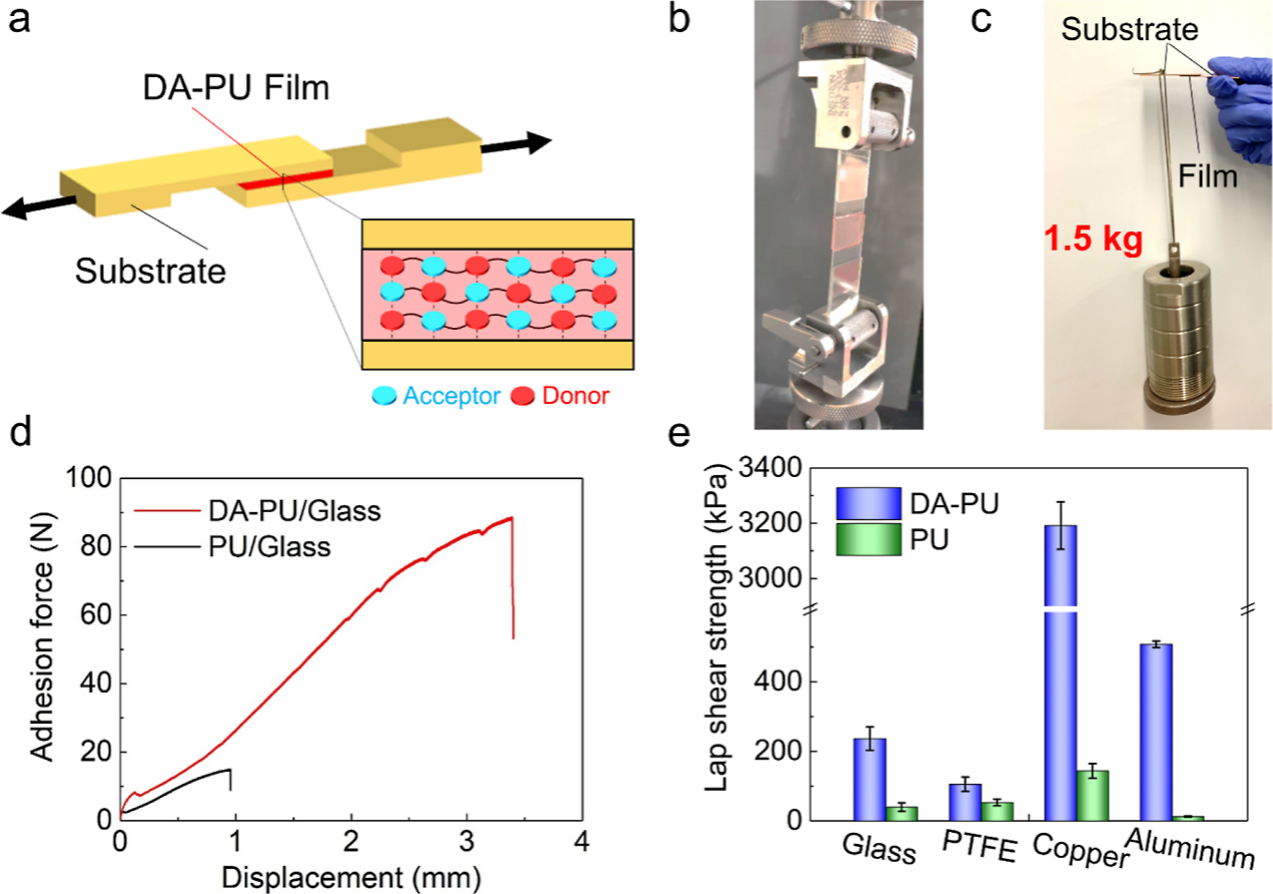
Adhesive properties. (a) Schematic of the sandwich specimen for the lap shear test and the aromatic group stacking mechanism in DA-PU. (b) Photograph of the DA-PU bonded glass sandwich specimen loaded in a universal testing machine. (c) Copper lap joint with DA-PU supporting 1.5 kg, that is, around 10^4^ times the film weight. (d) Adhesion force–displacement curves for glass lap joints. (e) Lap shear strengths of DA-PU and PU films on glass, PTFE, copper, and aluminum plates. Error bars represent standard deviation from three separate experiments (*p* < 0.05).

Lap shear tests with single lap joints were then carried out on a universal testing machine at room temperature (see Figure 3a,b; see details in the Supporting Information). The high adhesive strength of DA-PU between two copper plates enabled it to carry a heavy iron cylinder of 1.5 kg (Figure 3c), which was 10^4^ times the weight of the DA-PU film. Furthermore, when using glass substrates, the lap joint bonded by the DA-PU exhibited significantly higher peak loads and failure displacement compared to a PU lap joint, as shown in Figure 3d. The adhesion enhancement through D–A self-assembly was further quantified by comparing the lap shear strengths of DA-PU and PU on different substrates (Figure 3e). There was 6.5- and 2-fold enhancement on glass substrates and PTFE, respectively, while an order of magnitude improvement was observed with metallic substrates. Aromatic groups stacking between donors and acceptors is the main reason resulting in such universal adhesion enhancements^30,31^ and the drastic increase on metals, in particular copper, could be attributed to the formation of complex (such as Cu–O–C or Cu–N^54^) between the acceptors and the metal.

### 3.4 Formulating Superhydrophobicity

**Figure 4:**
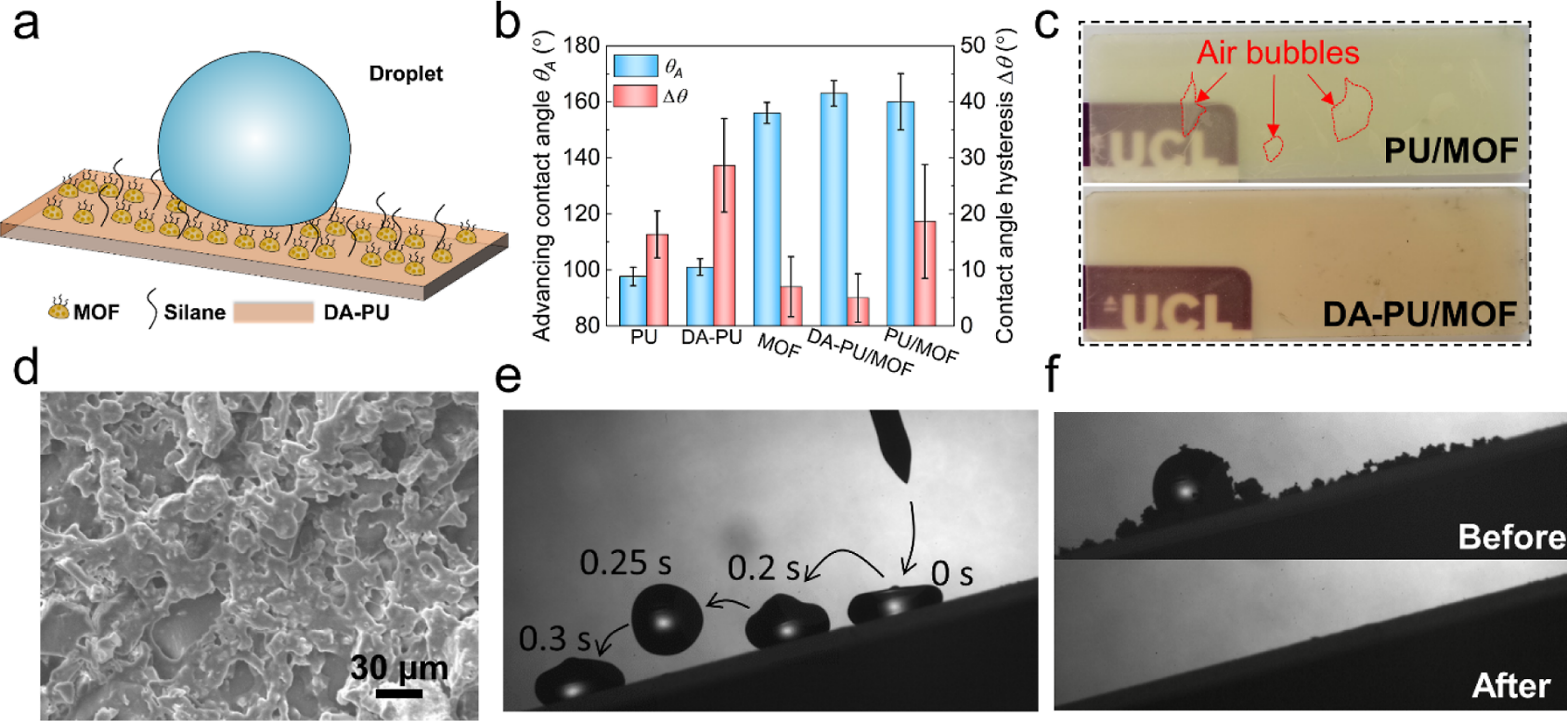
Surface wettability. (a) Schematic of the DA-PU/MOF SHS. (b) Water advancing contact angles (θ_A_) and hysteresis (Δθ) of different coatings. Error bars represent standard deviation from three separate experiments (*p* < 0.05). (c) Photos of PU/MOF and DA-PU/MOF coating on glass. (d) Morphology of DA-PU/MOF. Scale bar: 30 μm. (e) Merged snapshots from high-speed recording of a water droplet bouncing off the DA-PU/MOF surface. (f) Self-cleaning process, showing removal of contaminants (carbon powder) by water droplets rolling off the surface.

Introducing hydrophobicity into PU with plenty of oxygen-containing groups is a challenge, and most studies resort to fluorinated PU, which is harmful to the environment and toxic.^55,56^ To fabricate a fluorine-free SHS, alkyl-functionalized MOF nanoparticles with a nanohierarchical morphology were employed. Spray-coated films of MOF nanoparticles and DA–PU mixture were treated by OTS to achieve superhydrophobicity (see Figure 4a). The advancing contact angle (θ_A_) and hysteresis (Δθ) of different coatings based on DA-PU and PU are shown in Figure 4b. The pure MOF coating was prepared by spraying an acetone suspension of MOF nanoparticles. The θ_A_ on pure DA-PU was 3° higher than on PU, which is probably due to the lower density of hydrophilic oxygen-containing groups (urethane groups) after introducing the aromatic groups. The superhydrophobicity was achieved by including the MOF nanoparticles and silanization. Immersion in the hexane solution during the process of silanization led to partial separation and the generation of bubbles underneath the PU/MOF coating, while the DA-PU/MOF coatings maintained uniformity, indicating better substrate adhesion (Figure 4c). A SEM image of the DA-PU/MOF surface is shown in Figure 4d. The MOF particles were uniformly distributed in DA-PU film, and their nanoroughness is apparent from Figure S8 in Supporting Information. A repetitive jumping of a falling water droplet on DA-PU/MOF surface inclined at 15° (Figure 4e and Movie S2), and removal of surface contaminants (carbon powder) by rolling water drops, confirm the self-cleaning nature of the surface (see Figure 4f and Movie S3).

### 3.5 Robustness and Self-Healing

**Figure 5:**
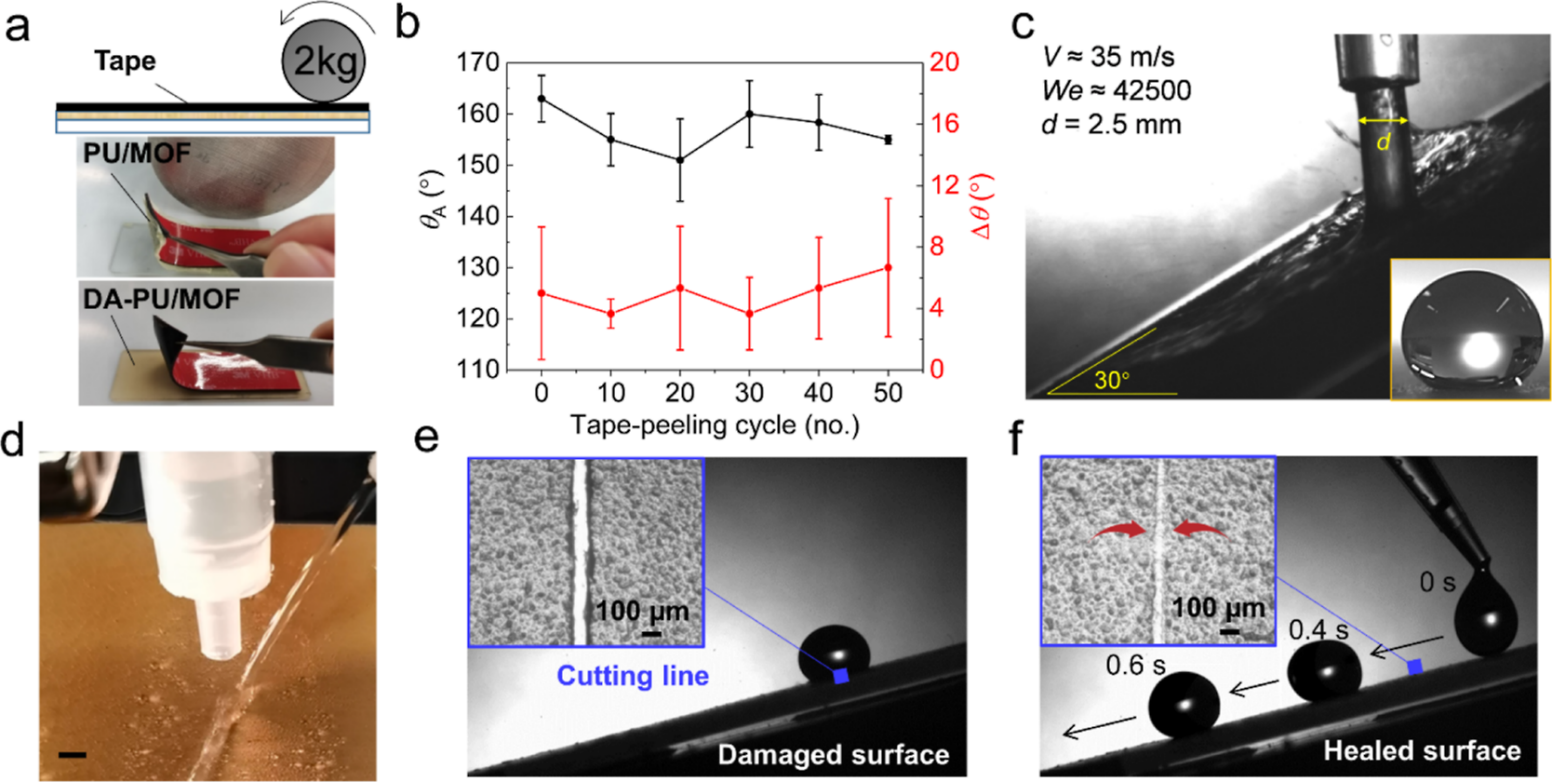
Robustness and the self-healing process of the DA-PU/MOF surface. (a) Top: Schematic of the tape peel test, middle: the PU based coating failing after one/two cycle, bottom: DA-PU/MOF coating remaining intact after 50 cycles. (b) Variation in the advancing water contact angle and hysteresis with tape peel cycles on DA-PU/MOF coating. Error bars represent standard deviation from three separate experiments (*p* < 0.05). (c) Water jet impacting at ∼35 m/s. Inset is droplet on the impacted site with a contact angle of 150°. (d) Photo of a low-speed water jet rebounding from the jet impact site. Scale bar: 2 mm. (e) Droplet stuck at the cut line on the surface. The inset shows optical microscope image of the cut. (f) Droplet mobility was recovered on the healed surface. Scale bar: 100 μm.

The robustness of the superhydrophobic DA-PU/MOF surface was tested using tape peel (Figure 5a,b) and high-speed liquid impact (Figure 5c) tests. No significant change in θ_A_ or Δθ was observed after 50 tape peel cycles using a high-tack 3M tape pressed by a 2 kg roller (Figure 5b). In contrast, owing to the low adhesive of PU with glass, the PU/MOF coating peeled completely after one or two cycles (see Figure 5a). Next, the liquid impalement resistance was tested using the high-speed water jet impact method.^7,32^ The DA-PU/MOF surface resisted impalement even after impact by jets >35 m/s, corresponding liquid Weber number ∼ 42,500 (see Methods in the Supporting Information). This was also confirmed by contact angles (the inset in Figure 5c shows a droplet) and rebounding a low-speed water jet at the impacted site (see Figure 5d and Movie S4). The excellent robustness is due to the high adhesion and mechanical properties of DA-PU, and the expected high impalement resistance of MOF nanoparticles is due to sub-nm scale pores. At high end of jet speeds, the impact location showed noticeable mechanical deformation (the cross-section of impacted surface in Figure S4 shows ∼300 μm indentation). The indent dimension is much smaller than the jet diameter (∼2.5 mm), due to the pressure peaks being localized near the point of impact (see ref (57)). The smaller size of the indentation should make it easier for self-healing to repair the surface.

Accidental damage or scratch of a superhydrophobic coating usually lead to local droplet pinning and loss of superhydrophobicity. These damaged spots can also form sites to initiate scaling, fouling, and ice formation by enabling nucleation. The self-healing feature of the DA-PU/MOF surface can avoid these. We demonstrate this by introducing a manual cut. Figure 5e shows a droplet stuck at the cutting line of the surface tilted at 15°. A notch with ∼100 μm width can be seen from the microscope image (Figure 5e inset). After heating the surface at 80 °C for 2 min, the droplet resumed rapid movement on the surface (Movie S5), and the notch had healed (Figure 5f). Additionally, the healed area was not smooth but rough, indicating that the creep of the DA-PU during the healing process must have also caused the migration of MOF nanoparticles. The simultaneous recovery of the coating and its roughness led to the repair of the superhydrophobicity of the surface.

### 3.6 Lubricant-Infusion and Anti-icing Application

**Figure 6:**
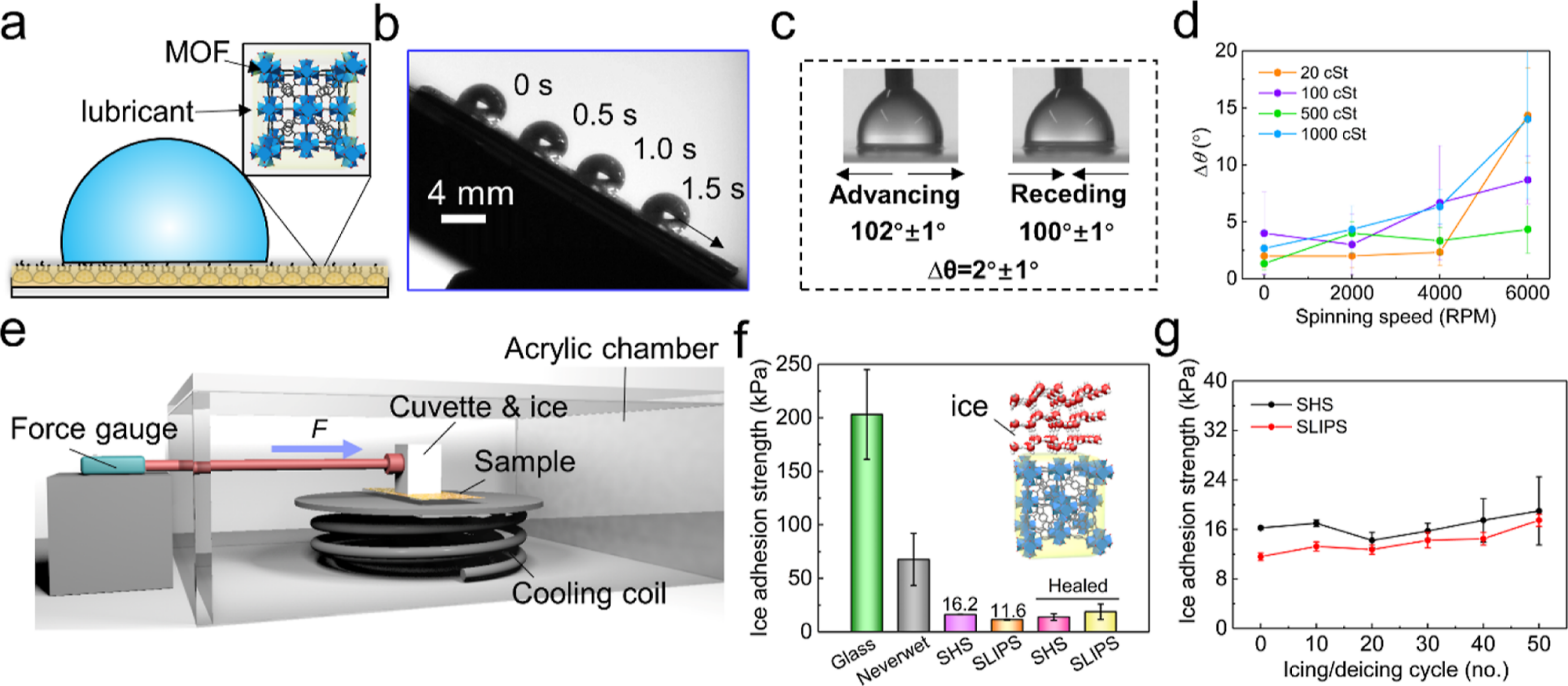
Anti-icing performance of SHS and SLIPS based on DA-PU/MOF. (a) Schematic showing our SLIPS prepared by infusing silicone oil in SHS. (b) Image of water droplet mobility with a shedding velocity of around 1.1 cm/s. (c) Photographs capturing advancing and receding angles of a water droplet on SLIPS. (d) Spinning stability of SLIPS infused with different viscosity silicone oils (20/100/500/1000 cSt) showing change in Δθ tested at 2000, 4000, and 6000 rpm. (e) Schematic of the ice adhesion measurement setup. (f) Ice adhesion strengths on different treatments measured at −20 °C. Inset shows the schematic of ice crystal above oil infused MOF. “Healed” indicates adhesion strengths measured on samples which had undergone self-healing of a notch (ice cuvette was placed right on top of the healed notch location). (g) Change in the ice adhesion strength of on our self-healing SHS and SLIPS for 50 icing/deicing cycles. Error bars represent standard deviation from three separate experiments (*p* < 0.05).

The porous nature of MOF nanoparticles in our coatings enables infusion of lubricants/oil to create SLIPS (see Figure 6a), which are known to be very effective at reducing ice adhesion and thereby facilitating passive, energy-efficient icing mitigation.^58,59^ Silicone oil (500 cSt) infused SLIPS enable excellent mobility of water droplets (Figure 6b) and slipperiness which was confirmed by measuring the dynamic wetting angles and hysteresis values of 2 ± 1° (see Figure 6c). 500 cSt silicone oil, showing the best stability at high-speed spinning among other viscosity oils (Figure 6d), was used for further anti-icing tests on SLIPS. The ice adhesion strength on different coatings was measured using a bench-top icing chamber, shown schematically in Figure 6e. Bare glass and commercial superhydrophobic coatings (NeverWet) were used as references. The ice adhesion strength on bare glass is comparable with the literature, for example, ∼220 kPa reported by Guo et al.^60^ and 276 kPa reported in Zhang et al.^61^ The lowest ice adhesion strength of 11.6 kPa was recorded on our SLIPS surface, which was lower than on the SHS (16.2 kPa) without the lubricant (shown in Figure 6f). Both of these ice adhesion strengths were much lower than that of the commercial SHS and also remained unchanged after self-healing from the notch. This superior ice adhesion characteristics should be attributed to the nanohierarchical morphology of the MOF particles, which reduces the adhesion and the interlocking of ice crystals with the texture.^32^ Surface texture, such as a smaller length scale (50–200 nm) and higher texture density, has been shown to have a significant effect on the SLIPS durability, as assessed through frost formation and ice-adhesion measurements.^62−64^ Compared with nanoscale porous surfaces (>50 nm) fabricated by traditional techniques, MOF-based coatings offer subnanometer pore size (<1 nm) and a dense texture (solid fraction ∼ 0.4^32^). This offers two advantages. First, the subnanometer size makes it hard for liquid to penetrate the pores, which leads to lower ice adhesion on the resulting SHS. Second, the pores apply superior capillary forces on oil to successfully combat frost and ice nuclei wicking. This adds to the stability of the oil on SLIPS in our work. Recent works have argued that SLIPS with finer textures should be more stable for anti-icing application as they retain the oil more strongly,^64,65^ which in turn offers low adhesion to ice. This better retention of oil and low interfacial forces between the oil and ice, explain the lower ice adhesion measured on our SLIPS. The cyclic ice adhesion measurements are shown in Figure 6g. After 50 icing/deicing cycles, the ice adhesion strength of SHS did not deteriorate significantly, while the ice adhesion strength of SLIPS increased slightly due to the loss of the lubricant entrained by ice. We suspect that the van der Waals interaction between the alkyl chains of MOF and silicone oil must be delaying the lubricant depletion, enabling a much higher number of icing/deicing cycles using our SLIPS than previously reported using hydrophilic NH_2_-UiO-66.^35^

## 4 Conclusions

In summary, we synthesized a supramolecular polyurethane, featuring donor–acceptor self-assembly which exhibited excellent self-healing capability and substrate adhesion characteristics. The electrostatic interaction and hierarchal hydrogen bonding between donor and acceptor units in the DA-PU enabled a notched coating to heal within 1 min under mild (80 °C) thermal activation. DA-PU also showed remarkable adhesion to glass, metals, and even PTFE. By blending the DA-PU with hydrophobic MOF nanoparticles and silanization, we obtained self-healing superhydrophobic coatings. The nanohierarchical and porous MOF particles could be infused with silicone oil to achieve SLIPS with excellent anti-icing characteristics, maintaining low ice adhesion even after 50 icing/deicing cycles. Our work offers insights in design and facile application of robust liquid-repellent coatings with damage tolerance and self-healing characteristics.

## Supporting Information Available

Details of robustness tests, self-healing test and simulation; synthesis route of DA-PU; ESP plots of donor and acceptor units; FTIR spectra of hydrophilic and hydrophobic MOFs, DA-PU and PU; PXRD graphs of hydrophilic and hydrophobic MOF nanoparticles; SEM images of the hydrophobic MOF powder; hydrophobicity of DA-PU/MOF coating at different MOF nanoparticle loadings (PDF)
In situ self-healing dynamic process recorded with a Peltier plate under the sample (MP4)
Repetitive jumping of a falling water droplet on the DA-PU/MOF surface inclined at 15° (MP4)
Removal of surface contaminants (carbon powder) by rolling water drops, confirming the self-cleaning nature of the surface (MP4)
Rebounding a low-speed water jet at the impacted site, showing that the DA-PU/MOF surface resisted impalement (MP4)
Droplet resuming rapid movement on the surface after heating it to 80 °C for 2 min (MP4)
