# Supplementary material for: Self-Healing, Robust, Liquid-Repellent Coatings Exploiting the Donor–Acceptor Self-Assembly
Source: ACS Appl Mater Interfaces. 2023 Feb 3;15(6):8699–708. doi: 10.1021/acsami.2c20636 (PMC9940105; doi:10.1021/acsami.2c20636)
Supplement: Supplementary file 3 — am2c20636_si_003.pdf [file am2c20636_si_003.pdf]

Supplementary Information

Self-healing, robust liquid-repellent coatings exploiting donor-accepter self-assembly

Jianhui Zhang[a,b], Vikramjeet Singh[a,b], Wei Huang[a,b], Priya Mandal[a,b], Manish K. Tiwari[a,b,]*

[a]Nanoengineered Systems Laboratory, UCL Mechanical Engineering, University College London, London, WC1E 7JE, UK

[b]Wellcome/EPSRC Centre for Interventional and Surgical Sciences, University College London, London, W1W 7TS, UK

***Corresponding author. Email: m.tiwari@ucl.ac.uk**

This file includes:

Materials and Methods

Figure S1 Synthesis route of DA-PU.

Figure S2 The electrostatic potential (ESP) of (a) donor unit and (b) acceptor unit.

Figure S3 FTIR of MOF and PU.

Figure S4 Self-healing property of DA-PU at different heating temperature.

Figure S5 PXRD graphs of NH_2-UiO-66 and modified MOF.

Figure S6 SEM images of hydrophobic MOF powder.

Figure S7 Hydrophobicity of DA-PU/MOF coating at different MOF nanoparticle loadings.

Figure S8 Velocity profile of high-speed jet impact.

Figure S9 Microscope image of DA-PU/MOF surface after jet.

Table S1 Comparison of liquid-repellent self-healing polymers from literature.

Supplementary movie 1-5.

SUPPLEMENTARY MATERIALS AND METHODS

Lap shear test for polymer adhesion

The adhesion strength of DA-PU and PU with different substrates (glass slide, PTFE, copper and aluminium plates) was measured by lap shear method using an universal testing machine (Instron, model 5969, 500 N load cell) at a constant cross-head speed of 0.5 mm/min (see Figure 3a). Polymer films with thickness around 0.5 mm were firstly prepared by casting a DMF solution and drying in an oven. The resulting films with area 25 mm × 15 mm were then placed on a substrate under test, followed by overlaying another substrate to form a test specimen for adhesion measurements. The specimens were heated at 80 °C for 1 h to bond the substrates with the polymer. Alignment sheets were attached to both ends of the specimen to reduce the bending moment during shear tests (Figure 3a). The lap shear strength was calculated by dividing the maximum load by the initial sandwich area and at least three samples tested for statistics.

Tape peel test

A strong bonding tape (3M™ VHBTM tape 5952, with an adhesion to steel value of 3,900 N/m) was used to test the adhesion of superhydrophobic coatings on glass slide with area 2.5 cm × 7.5 cm (shown in Figure 5a). The tape was applied by rolling a 2 kg steel roller on the tape twice, then waiting for 60 s and followed by peeling off the tape. The process of tape application and peel-off was considered as one cycle. The steps were repeated with contact angle measurements following each cycle. A fresh piece of tape was used for each cycle.

Spinning stability test

Retention of different viscosity lubricants (silicone oil 20, 100, 500, and 1000 cSt) by DA-PU/MOF was tested using a spinning stability test. After infusion, the samples with area 20 mm × 20 mm were tilted at 90° for one hour and then spun at 1000 RPM to get rid of excessive lubricant, followed by contact angle measurement. This was denoted "0 RPM" measurement to indicate lack of excess spinning stability test after substrate preparation. Substrates were then spun at 2000, 4000 and 6000 RPM for 1 min with contact angle measurement each time after spinning.

Statistical analysis

One-way ANOVA and Dunnet's multiple comparison tests were applied for intergroup comparisons using the least significance difference tests in R software. The intergroup comparisons were performed on data including contact angle hysteresis, ice adhesion strength and lap shear strength. A p-value less than 0.05 was considered statistically significant. All data were reported as mean and standard deviation from three separate measurements.

Density function theory simulation

Although donor and acceptor self-assembly has been widely studied in the area of organic electronics [1], its use in self-healing polymers is still new [2]. The enhanced hydrogen bonding between donor and acceptor unit has been studied through FTIR in Figure 1f and Ref. [2]; however, the physical interactions at atomic level and their contribution to the self-healing remain unclear. Density function theory (DFT) is a powerful first principle tool to simulate intermolecular interactions with high precision. The initial donor and acceptor unit structure was built in the Gaussian View program, and then optimised by DFT at B3LYP/6-311G basis set in the Gaussian 16 program [3, 4]. The interaction energy $E_{interaction}$ was calculated with respect to the isolated donor and acceptor unit and corrected for the basis-set superposition error by the counterpoise correction method as follow [5]:

$$E_{interaction} = E_{DA} - E_D - E_A + E_{BSSE} \quad \text{(S1)}$$

where E_{DA}, E_D, E_A and E_{BSSE} are energies of donor-acceptor complex, donor unit, acceptor unit and basis set superposition error, respectively.

To understand the electrostatic interactions between the donor and acceptor unit, electrostatic potential (ESP) analysis was undertaken. The single point energy was calculated by def2-TZVP basis set with higher accuracy. The isopotential surfaces of ESP, including single donor, acceptor, and their complex, was analysed through Multiwfn program and are shown in Figure S1 and Figure 1d [6].

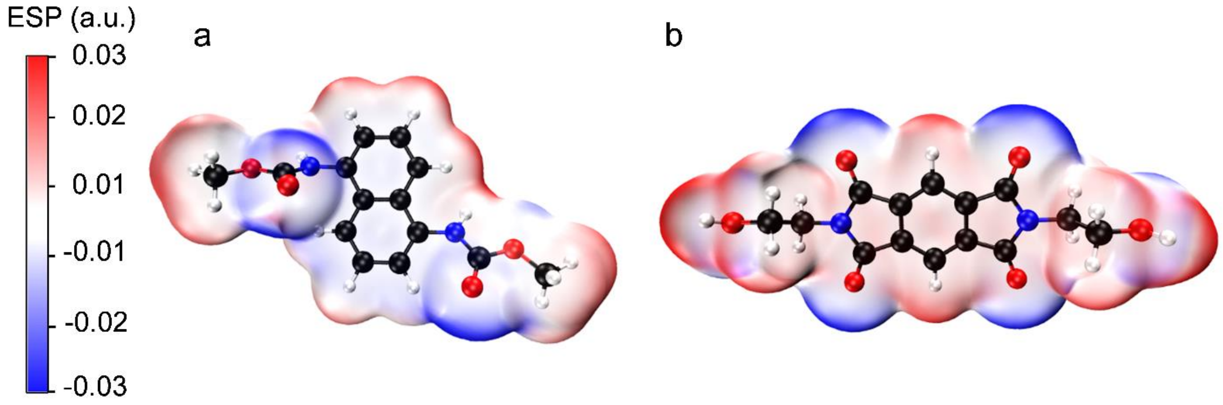

Figure S1 The electrostatic potential (ESP) of (a) donor unit and (b) acceptor unit. Coloured balls represent different atoms: white is H, red is O, blue is N, black is C. The negative surface potential is indicated in blue while the positive surface potential is indicated in red.

Self-healing test

The self-healing process was observed after cutting the middle portion of the coating with a 400-µm thick blade. The notch recovery at 60 °C and 80 °C was recorded using a digital microscope (Keyence, VHX-7000) at 500× magnification, shown in Figure 2. The notch depth of films was kept at ~400 µm. An *in situ* self-healing dynamic process in the range of 40 ~ 90 °C was also recorded with a Peltier plate under the sample, shown in Figure S2 and Supplementary movie 1. The self-healing of the surface was evaluated by assessing droplet mobility on surface. The notch in the film initially prevented the droplet from rolling down at an inclination angle of 15° (See Supplementary movie 5). The droplet motion was recorded using a Phantom V411 high-speed camera fitted with a macro lens. Then the surface was heated at 80 °C for 2 min to heal the notch. The coatings sample was also inspected under a GXML3200B compound microscope at 40× magnification and photos were taken with a DCC1545M CMOS camera (Thorlabs) attached to the microscope (see Figure 5e and 5f).

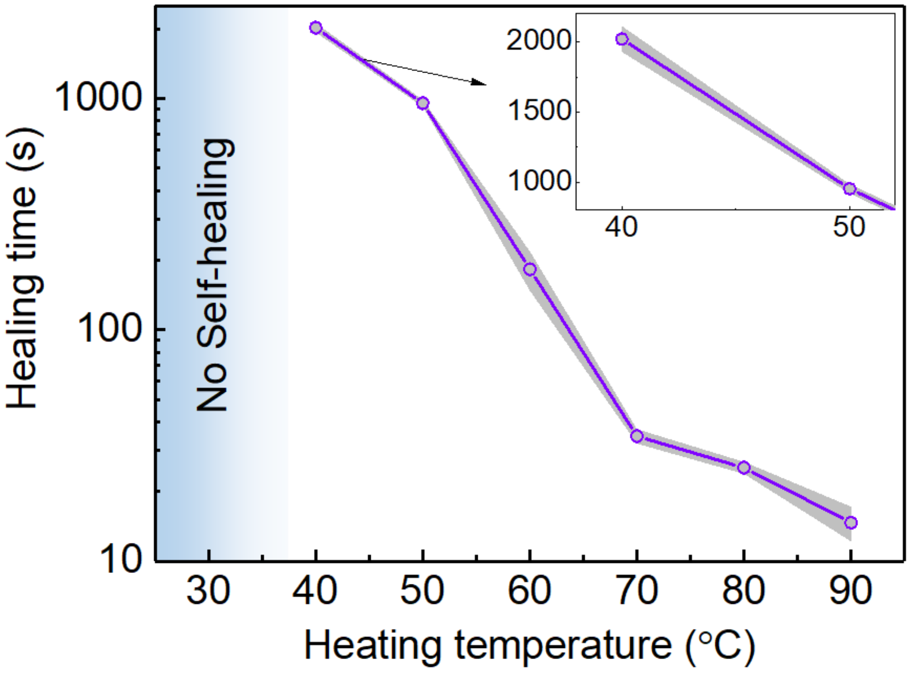

Figure S2 Self-healing capability of DA-PU at different temperatures. The healing time was determined by the time the sample needed to be on the hot plate for a notch (~100 µm) to heal and not be visible any longer under an optical microscope. No self-healing was observed at room temperature; however, it could heal it could self-heal efficiently when exposed >40 °C. The healing was faster at higher temperatures (>80 °C) but mainly limited by the heat transfer from Peltier. The shaded region represents standard deviation from three separate experiments ($p < 0.05$).

Table S1 Comparison of liquid-repellent self-healing polyurethane from literature.

Self-healing mechanism	Self-healing temperature (°C)	Healing time (min)	Applications	References
Donor-acceptor self-assembly	60-80	< 5	Anti-icing	Current work
Diels-Alder chemistry	150	900-3000	-	[7]
Disulfide bonding	25-60	9-500	Electronic skin	[8]
	70	60	Anti-corrosion	[9]
	60	120	Sensing	[10]
Phenolic urethane chemistry	130	40	Self-cleaning	[11]
Multiple hydrogen bonding	Room temperature	30	Cathodic Protection	[12]
		1440-5760	Anti-icing	[13]
		~2880	Anti-icing	[14]

Jet impact test

A custom pneumatic setup, similar that reported elsewhere [15], connected to an electronic pressure valve and nitrogen gas cylinder were used for jet impact test and the piston pressure kept under 12 bar. The speed of 2.5-mm jet was calculated from motion of the piston (Figure S3) and the related geometric parameters such as nozzle diameter using following equation:

$$\pi d_s^2 \, \Delta h/4 = \pi d_n^2 V_{jet} \, \Delta t/4 \quad \text{(S2)}$$

where Δt is the time, Δh is the distance, d_s is the cylinder diameter, d_n the nozzle (jet) diameter and V_{jet} the jet speed.

The maximum jet speed reached in our experiments (~35 m/s) was determined by averaging the maximum speeds in different tests, as shown in Figure S3. The corresponding liquid Weber number (We_l=$\rho V^2 \mathrm{d}_n/\gamma$) for the jet calculated was ~42,500. Aluminum plates with area 5 cm × 5 cm was coated with a thick layer (~ 500 μm) of DA-PU/MOF, followed by the jet impact. The surface showed a small indentation (Figure S4) after jet impact. Water droplets rolled off and low-speed jet rebounded from the impact spot, suggesting a good liquid impalement resistance.

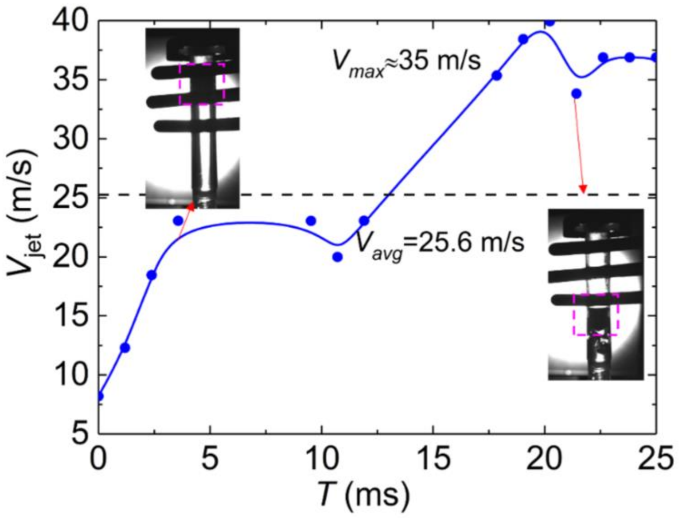

Figure S3 Velocity profile of high-speed jet impact. Inset photos show the motion of the piston. Air cavity was generated from the compression of piston, leading to turbulent multiphase jet in the end.

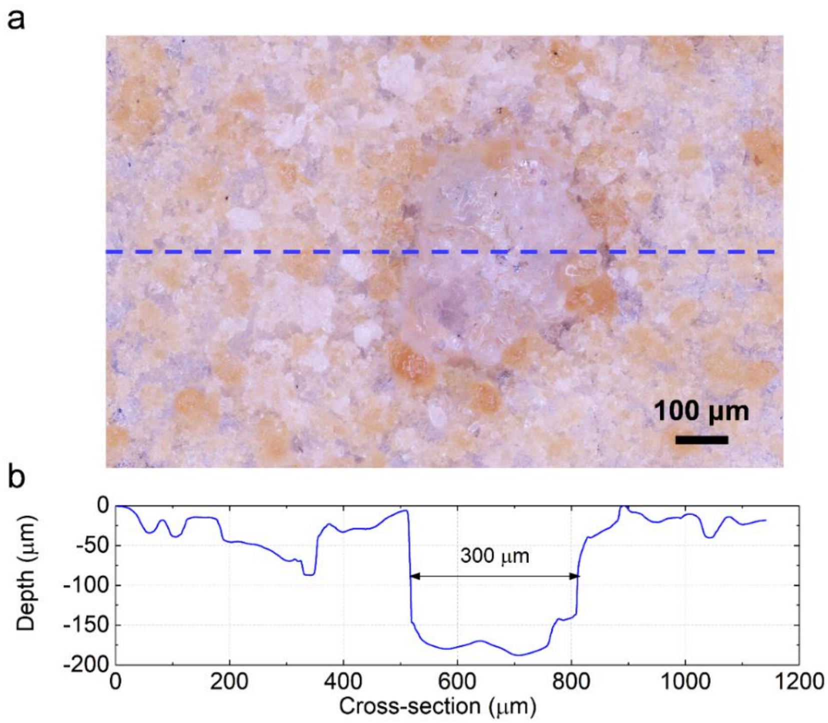

Figure S4 Microscope image of DA-PU/MOF surface after jet. (a) The optical microscope image of DA-PU/MOF surface after repeated impact (3 times) with indentation in centre. (b) The profile of the cross-section was measured by images decomposition of 3D microscope. The ~300 µm indentation was generated by the jet impact with ~ 2.5 mm diameter. It should be easier for the self-healing of the smaller size indentation to repair the surface.

Synthesis of DA-PU

Figure S5 below depicts the steps in DA-PU synthesis (described in section 2.3 of the main paper).

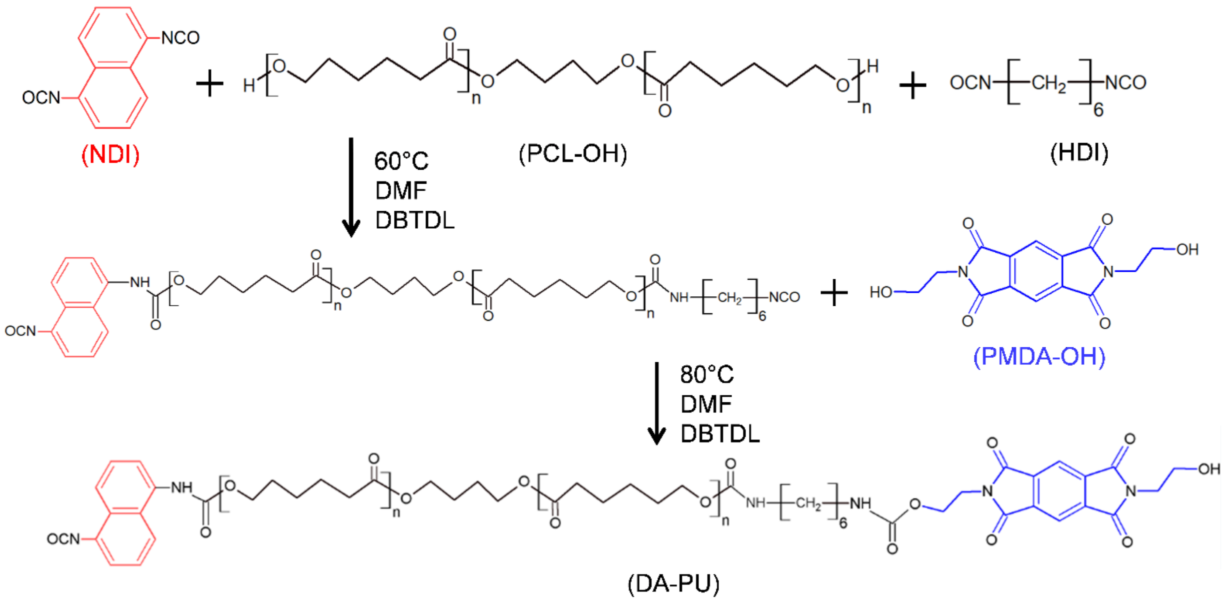

Figure S5 Synthesis route of DA-PU.

Characterization

Figure S6a shows the FTIR absorption peak of stretching of the carbonyl (C=O) bond in the amide group around 1690 cm^{-1}, suggesting successful synthesis of hydrophobic alkyl-UiO-66. The FTIR spectra of DA-PU and PU is presented in Figure S6b. The peak at 1680 cm^{-1} is attributed to the associated hydrogen bonds between donor and acceptor units.

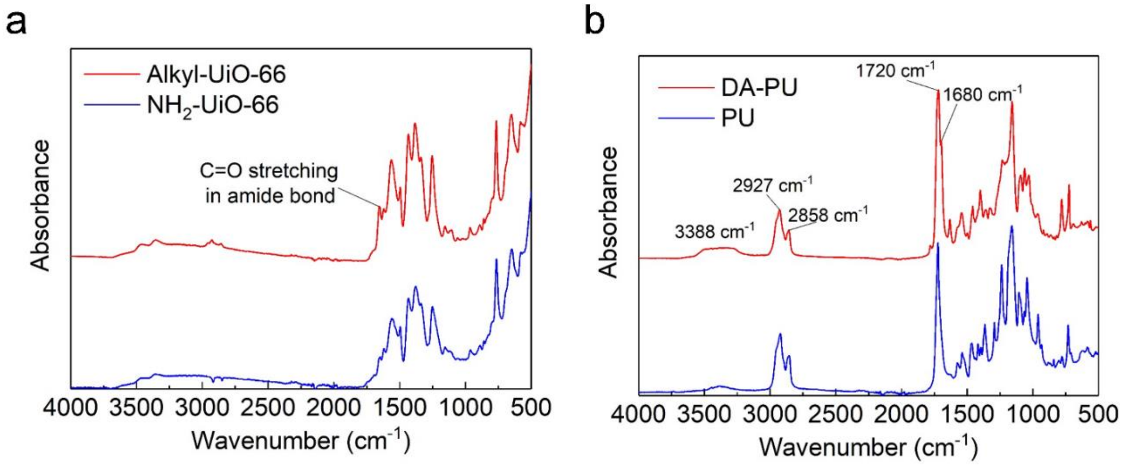

Figure S6 FTIR of MOF and PU. (a) FTIR spectra of hydrophilic MOF (NH_2-UiO-66) and hydrophobic alkyl-UiO-66. The peaks of red line at 1690 cm^{-1} can be attributed to stretching of the carbonyl (C=O) bond in the amide group, confirming the successful covalent linkage of alkyl chains to the amino functional group of the hydrophilic MOF. (b) FTIR spectra of DA-PU and PU. Characteristic bonds of the hydroxyl and carbonyl groups for both DA-PU and PU were observed in the peaks 3388 and 1720 cm^{-1}. The symmetric

and non-symmetric stretching of the C-H bond with carbonyl was obtained between 2927 and 2858 cm^{-1}. The characteristic peak at 1680 cm^{-1} is attributed to the associated hydrogen bonds.

The consistency of the main diffraction peaks observed in the PXRD spectra of two MOFs (Figure S7) suggests that the side chains substituted through post-functionalization did not change the original crystal structure and porosity feature (subnanometer pores) of MOF.

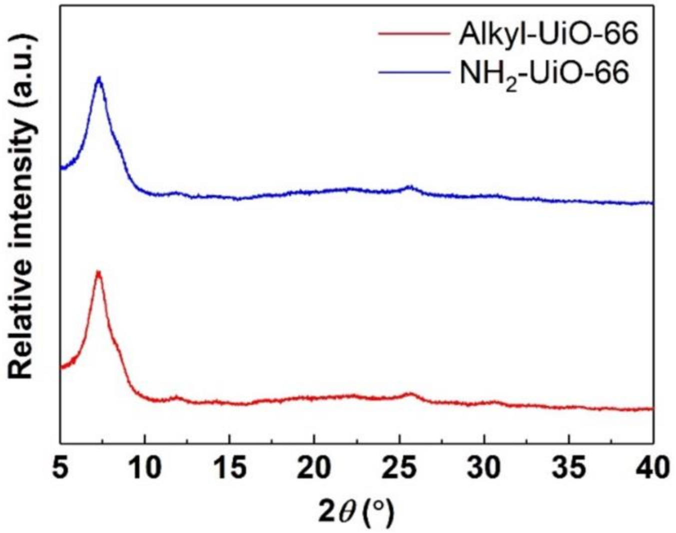

Figure S7 PXRD graphs of NH_2-UiO-66 and modified MOF (Alkyl-UiO-66) confirming the successful synthesis and post-synthetic modifications.

The SEM images of alkyl-UiO-66 in Figure S8 show the morphology of MOF nanoparticles. The nanohierarchical morphology can be inferred from the SEM images (which confirm particle sizes to be at several hundred nanometer scale) and the PXRD spectra in Figure S7 (which confirm the presence of subnanometer scale MOF pores).

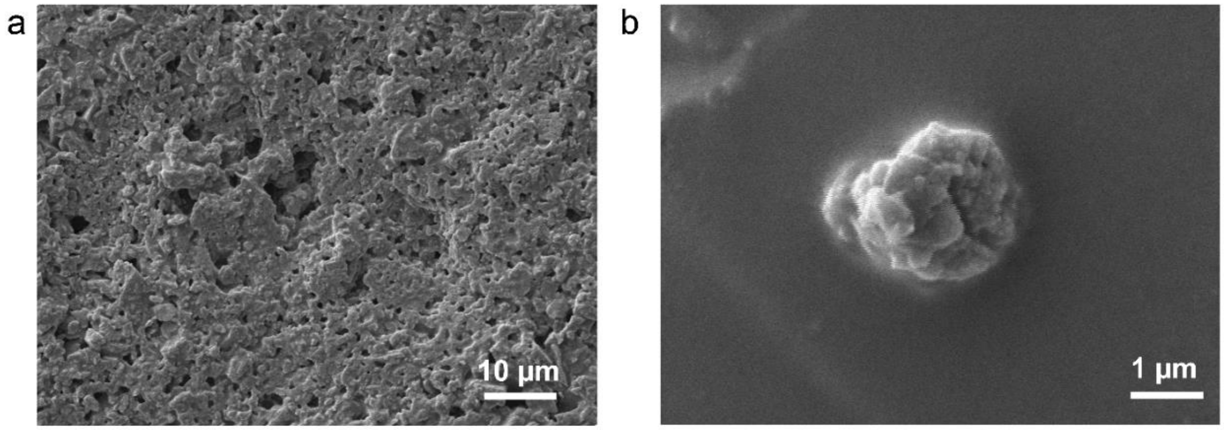

Figure S8 SEM images of hydrophobic MOF powder, confirming the nanohierarchical morphology (several hundred nm particles and subnanometer pores).

MOF loading optimization

DA-PU nanocomposites with varying MOF nanoparticle concentration were prepared to determine the effect of MOF loadings on liquid repellence. Figure S9 shows the results, with advancing contact angle showing rising trend up to 50% loading and then plateauing. The mean hysteresis remains <10° for all concentrations.

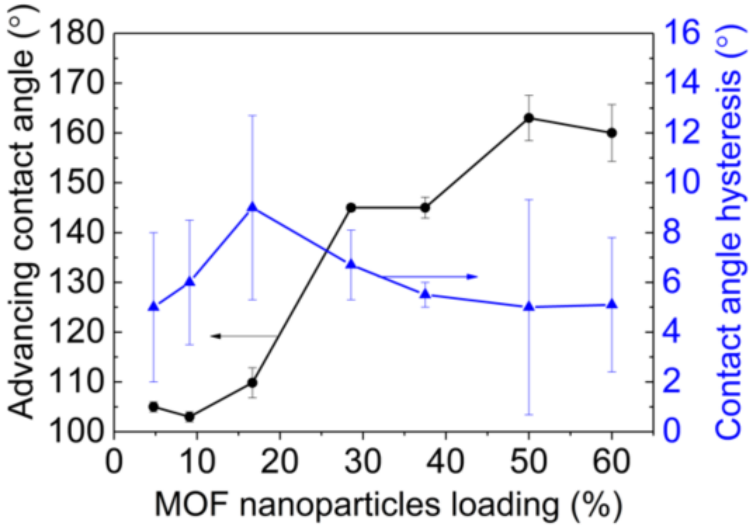

Figure S9 Hydrophobicity of DA-PU/MOF coating with different MOF nanoparticle loadings. The advancing contact angles of the nanocomposite coatings increased with MOF loading up to 50%. Beyond 50%, both contact angle and hysteresis remained unchanged. Error bars represent standard deviation from three separate experiments ($p < 0.05$).

Captions for supplementary movies

Supplementary movie 1: The dynamic self-healing process of notched DA-PU film at 60 °C and 80 °C recorded using a digital microscope with a Peltier plate underneath. The Peltier plate (heating from bottom) induced a bottom-to-top self-healing process due to inevitable thermal gradient, which is different from the uniform healing (marked by cut interfaces approaching each other) under uniform temperature field of an oven shown in Figure 2a and 2b.

Supplementary movie 2: The droplets bounced easily above DA-PU/MOF superhydrophobic surface (the movie captures an example where the surfaces is titled at 15°).

Supplementary movie 3: Water drops rolling off on DA-PU/MOF surfaces, easily removed contaminants (carbon powder) confirming self-cleaning property.

Supplementary movie 4: A water jet with nozzle diameter 2.5 mm impacted on DA-PU/MOF surface at ~35 m/s velocity recorded by high-speed camera. After high-speed jet impact, water droplets rolled off and low-speed jets rebounded from the impact spot.

Supplementary movie 5: Droplet mobility damaged after the surface cut by knife and recovered after healing.

References

[1] Marszalek, T.; Li, M.; Pisula, W. Design Directed Self-assembly of Donor–acceptor Polymers. *Chem. Commun.* **2016**, 52(73), 10938-10947, DOI: 10.1039/C6CC04523E

[2] Ying, W. B.; Wang, G.; Kong, Z.; Yao, C. K.; Wang, Y.; Hu, H.; Li, F.; Chen, C.; Tian, Y.; Zhang, J.; Zhang, R.; Zhu, J. A Biologically Muscle-inspired Polyurethane with Super-tough, Thermal Reparable and Self-healing Capabilities for Stretchable Electronics. *Adv. Funct. Mater.* **2021**, 31(10), 2009869, DOI: 10.1002/adfm.202009869

[3] Becke, A.D. Density-Functional Thermochemistry .3. The Role of Exact Exchange. *J. Chem. Phys.* **1993**. 98(7), 5648-5652, DOI: 10.1063/1.464913

[4] Lee, C. T.; Yang, W. T.; Parr, R. G. Development of the Colle-salvetti Correlation-energy Formula into a Functional of the Electron-density. *Phys. Rev. B* **1988**, 37(2), 785-789, DOI: 10.1103/PhysRevB.37.785

[5] Boys, S. F.; Bernardi, F. J. M. P. The Calculation of Small Molecular Interactions by the Differences of Separate Total Energies. Some Procedures with Reduced Errors. *Mol. Phys.* **1970**, 19(4), 553-566, DOI: 10.1080/00268977000101561

[6] Lu, T.; Chen, F. Multiwfn: a Multifunctional Wavefunction Analyser. *J. Comput. Chem.* **2012**, 33(5), 580-592, DOI: 10.1002/jcc.22885

[7] Behera, P. K.; Mondal, P.; Singha, N. K. Self-healable and Ultrahydrophobic Polyurethane-POSS Hybrids by Diels–alder "Click" Reaction: a New Class of Coating Material. *Macromolecules* **2018**, 51(13), 4770-4781, DOI: 10.1021/acs.macromol.8b00583

[8] Ying, W. B.; Yu, Z.; Kim, D. H.; Lee, K. J.; Hu, H.; Liu, Y.; Kong, Z.; Wang, K.; Shang, J.; Zhang, R.; Zhu, J.; Li, R. W. Waterproof, Highly Tough, and Fast Self-healing Polyurethane for Durable Electronic Skin. *ACS Appl. Mater. Interfaces* **2020**, 12(9), 11072-11083, DOI: 10.1021/acsami.0c00443

[9] Li, C.; Wang, P.; Zhang, D.; Wang, S. Near-infrared Responsive Smart Superhydrophobic Coating with Self-healing and Robustness Enhanced by Disulfide-bonded Polyurethane. *ACS Appl. Mater. Interfaces* **2022**, 14(40), 45988-46000, DOI: 10.1021/acsami.2c08496

[10] Chen, K.; Liu, H.; Zhou, J.; Sun, Y.; Yu, K. Polyurethane Blended with Silica-nanoparticle-modified Graphene as a Flexible and Superhydrophobic Conductive Coating with a Self-healing Ability for Sensing Applications. *ACS Appl. Nano Mater.* **2022**, 5(1), 615-625, DOI: 10.1021/acsanm.1c03414

[11] Naveed, M.; Rabnawaz, M.; Khan, A.; Tuhin, M. O. Dual-layer Approach toward Self-healing and Self-cleaning Polyurethane Thermosets. *Polymers* **2019**, 11(11), 1849, DOI: 10.3390/polym11111849

[12] Sun, W.; Luo, N.; Liu, Y.; Li, H.; Wang, D. A New Self-Healing Triboelectric Nanogenerator Based on Polyurethane Coating and Its Application for Self-Powered Cathodic Protection. *ACS Appl. Mater. Interfaces* **2022**, 14(8), 10498-10507, DOI: 10.1021/acsami.2c00881

[13] Zhuo, Y.; Håkonsen, V.; He, Z.; Xiao, S.; He, J.; Zhang, Z. Enhancing the Mechanical Durability of Icephobic Surfaces by Introducing Autonomous Self-healing Function. *ACS Appl. Mater. Interfaces* **2018**, 10(14), 11972-11978, DOI: 10.1021/acsami.8b01866

[14] Zhuo, Y.; Xiao, S.; Håkonsen, V.; Li, T.; Wang, F.; He, J.; Zhang, Z. Ultrafast Self-healing and Highly Transparent Coating with Mechanically Durable Icephobicity. *Appl. Mater. Today* **2020**, 19, 100542, DOI: 10.1016/j.apmt.2019.100542

[15] Peng, C. Y.; Chen, Z. Y.; Tiwari, M. K. All-organic Superhydrophobic Coatings with Mechanochemical Robustness and Liquid Impalement Resistance. *Nature Mater.* **2018**, 17(4), 355-360, DOI: 10.1038/s41563-018-0044-2
